# Development of Plant Protein Derived Tri Angular Shaped Nano Zinc Oxide Particles with Inherent Antibacterial and Neurotoxicity Properties

**DOI:** 10.3390/pharmaceutics14102155

**Published:** 2022-10-10

**Authors:** Tianyu Hou, Siva Sankar Sana, Huizhen Li, Xin Wang, Qinqin Wang, Vijaya Kumar Naidu Boya, Ramakrishna Vadde, Raj Kumar, Divya Vishambhar Kumbhakar, Zhijun Zhang, Narsimha Mamidi

**Affiliations:** 1 School of Chemical Engineering and Technology, North University of China, Taiyuan 030051, China; 2 Jinzhong Institute of Industrial Technology and Innovation, North University of China, Jinzhong 030600, China; 3 Department of Material Science and Nanotechnology, Yogi Vemana University, Kadapa 516005, India; 4 Department of Biotechnology and Bioinformatics, Yogi Vemana University, Kadapa 516005, India; 5 Department of Pharmaceutical Sciences, University of Nebraska Medical Center, Omaha, NE 68105, USA; 6 School of Medical Sciences, University of Hyderabad, Hyderabad 500046, India; 7 Department of Chemistry and Nanotechnology, School of Engineering and Science, Tecnologico de Monterrey, Monterrey 64849, Mexico

**Keywords:** biosynthesis, N-ZnO Ps, HR-TEM, antibacterial activity, anticancer activity

## Abstract

The synthesis of nanometer-sized metallic nanoparticles utilizing bio-sources is one of the most cost-effective and ecologically friendly approaches. Nano-zinc oxide particles (N-ZnO Ps) were made using a simple green synthesis method using an aqueous zinc nitrate salt and *Perilla frutescens* crude protein as a protecting and reducing agent in the current work. UV-visible (UV-vis) spectrophotometry, X-ray diffraction (XRD), X-ray photoelectron spectroscopy (XPS), Fourier transform infrared spectroscopy (FT-IR), scanning electron microscopy (SEM), (energy dispersive x-ray spectroscopy) EDX and high-resolution transmission electron microscopy (HR-TEM) were used to characterize the synthesized N-ZnO Ps. A distinctive UV-vis absorption peak was observed at 370 nm due to N-ZnO Ps. The SEM and HR-TEM pictures revealed N-ZnO Ps with a triangular form. The XRD pattern indicated the wurtzite structure of N-ZnO Ps. Nanoparticles exhibited a zeta potential of −11.3 mV. The antibacterial activity of N-ZnO Ps was tested against *Escherichia coli* (*E. coli*) and *Klebsiella pneumoniae* (*K. pneumonia*) microorganisms. The N-ZnO Ps were non-toxic to HMC-3 human normal brain microglia cells; however, they exhibited a potential cytotoxic effect on the LN-18 human brain glioblastoma cell line. These results indicate that N-ZnOPs can act as promising antibacterial and anticancer treatments in the prevention of Glioblastoma.

## 1. Introduction

**Figure 1:**
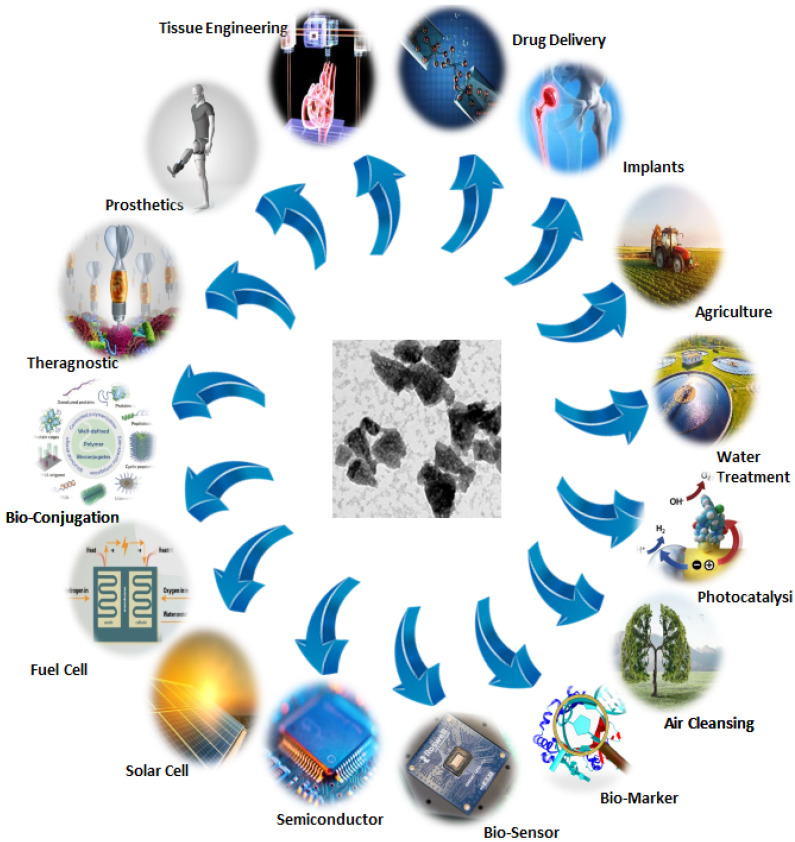
Applications of N-ZnO Ps.

In recent years, the development of environmentally friendly methods for producing metal oxide nanoparticles has developed into a significant area of nanotechnology. Metal oxide nanoparticles have immense potential applications in the field of nanoscience, because of their semiconducting, antibacterial, antifungal, wound healing, and UV-filtering properties [1]. Nanotechnology has extensive uses because it is easy to change crucial structures of substances at the nanoscale to attain properties in modern research. Additionally, it has received much attention due to the qualities that help its application in various fields, including sunscreens, medications, paints, and beauty products [2]. Green nanotechnology plays the most important role in modern scientific research, due to its attractive and unique novel qualities for the design of materials. Among metal oxide nanoparticles, zinc oxide (ZnO), has received interest from researchers throughout the world. ZnO nanoparticle has demonstrated excellent antioxidant potential and antimicrobial and anti-cancerous agents due to its 3.3 ev low band gap at room temperature and potential for reactive oxygen molecule (ROS) [3]. The commercial use of nano zinc-oxide particles (N-ZnO Ps) is included as food packing material due to its antimicrobial nature [3], in the rubber industry as an activator [4], in the semiconductor industry [5], in cement as an additive material [6], and as a UV protective agent [7], etc. Zinc is an essential component of eukaryotic cells and is found in numerous enzymes and proteins that are involved in a variety of metabolic processes, including the pathway that uses glucose. The general applications of N-ZnO Ps are shown in Figure 1.

Various chemical and physical processes are used for the fabrication of N-ZnO Ps in bulk amounts, including polyol [8], direct precipitation [9], pulsed laser deposition [10], sol-gel [11], and microwave-assisted combustion [12]. Synthesizing metal nanoparticles using high-energy chemical and physical processes involves hazardous and aggressive substances such as reducing and capping agents [13]. As a result, leftover harmful substances and by-products may be adsorbed on the surface of nanoparticles. Plants, unlike bacteria, can create huge quantities of nanoparticles and can serve as long-lasting and ecologically benign repositories.

Moreover, when plant sources are used, the synthesized nanoparticles are easily adaptable at normal pressure and room temperature with a great capacity for rescaling, and they are bio-compatible and non-hazardous [14]. Variable co-enzymes and phytoconstituents soluble in water (e.g., quinones, flavonoids, amino acids, reducing sugars, polyphenols, proteins, catechins, and terpenoids) have been used for synthesizing nanoparticles using plant metabolites as capping agents, as well as reducing agents, to prepare high surface-area metal nanoparticles [15,16]. Therefore, they are considered green sources to prepare preferred morphological natural nanoparticles. Plant extract-mediated nanoparticle synthesis is preferred because it is bio-friendly, simple, cost-effective, and safe for biomedical applications [17]. 

Green nanoparticle production is an extracellular bio-synthetic approach that uses phytochemicals as reducing and stabilizing agents. Bioreduction converts metal ions and metal oxides into zero valence metal nanoparticles (NPs) using phytochemicals produced by plants, such as polyphenolic compounds, alkaloids, polysaccharides, amino acids, vitamins, and terpenoids [18]. Metal nanoparticle production is presently being refined by scientists [3,4,5,6].

N-ZnOPs can be synthesized from plant parts such as leaves, stems, roots, fruits, and seed proteins because they produce specific phytochemicals. Extracts of plants contain secondary plant compounds that act as reducing agents and capping agents. A variety of phytochemicals, including polyphenolic compounds, alkaloids, polysaccharides, amino acids, vitamins, and terpenoids, reduce metal ions or metal oxides during bioreduction [18]. The medicinal plant *Perilla frutescens* L. (Labiatae) is a traditional crop used in Asia for its seeds, essential oils, and medicinal purposes [19]. It is anti-inflammatory, anti-allergic, detoxicant, anti-tussive, antibiotic, and antipyretic, and it treats digestive issues [20]. A by-product of the oil extraction from *P. frutescens* seeds has received little attention. An extensive study on *P. frutescens* seed protein is required for optimal utilization. In this study, biogenic nanoparticles of zinc oxide with a triangular shape were synthesized using an aqueous zinc nitrate salt solution and *P. frutescens* crude protein as a protecting and reducing agent for the first time. An evaluation of the produced N-ZnO Ps using UV-vis, FT-IR, HR-TEM, SEM with EDX, XRD pattern, zeta potential, l, and XPS was conducted. In addition to antibacterial properties, N-ZnO Ps were also tested for anticancer effects. To understand cancer biology and intelligently design NPs for better clinical use, clinicians, biologists, and material scientists need to collaborate more closely. Developing smart green NPs that select for cancer cells with more accuracy and are less toxic to normal cells may be possible through a dynamic collaboration.

## 2. Materials and Methods

### 2.1. Reagent and Materials

This research employed two cell lines (HMC-3 normal brain microglia and LN-18 human brain glioblastoma) cultured at 37 °C in 5% CO_2_ using Dulbecco’s Modified Eagle’s Medium (DMEM) with 10% fetal bovine serum (FBS), penicillin/streptomycin and amphotericin B solutions, respectively, in the following concentration (100 µg/mL:100 µg/mL:2.5 µg/mL). The zinc nitrate was supplied by Merck, Mumbai, India.

### 2.2. Preparation of Crude Perilla Protein 

We have prepared *Perilla frutescens* protein, as explained in our published work [21]. In brief, perilla seeds were harvested in good condition, dried for 2 h at 37 °C, ground, and sieved using a 60-mesh sieve. The flour was then defatted in an extractor using three times the amount of petroleum ether for 30 min. The defatted flour was produced after three repetitions and one hour at 50 °C. We used a 1:10 biomass-to-volume ratio suspension in pH 10 water. After 60 min at 55 °C, the alkali-aided solubilized proteins were collected. Adding hydrochloric acid and centrifuging at 10,000 rpm for 20 min at 4 °C produced isoelectric protein precipitation.

### 2.3. Biosynthesis of ZnO Nanoparticles

The N-ZnO Ps were synthesized using the sol-gel technique [22] with minor modifications. A solution of 20 mL distilled water containing a known amount of zinc nitrate was agitated for 30 min. Then, the protein solution was put into an oil bath with zinc nitrate solution and kept at pH 11 with 1N NaOH solution. The oil bath was held at 60 °C and stirring for 5 h yielded a light-brown resin. The finished product was rinsed three times with distilled water and calcined for three hours at 400 °C to obtain N-ZnO Ps. Finally, the white powdered N-ZnO Ps were labeled and kept for later use.

### 2.4. Characterization of ZnO Nanoparticles

An aliquot of N-ZnO Ps suspension in Millipore water used for UV-vis absorption spectroscopy was used to identify the optical properties of synthesized nanoparticles at wavelengths ranging from 300–600 nm, employed by UV-visible spectrometer, LAB INDIA, model-3092, Mumbai, India. The FT-IR spectrum was obtained from Perkin Elmer, model two, UK, in the range of 4000–400 cm^−1^ by the KBr pellet technique at room temperature. It is used to identify the chemical constituents present in synthesized NPs and to examine the functional groups of biomaterials. To determine the phase purity and crystalline nature of N-ZnO Ps, the XPS analysis was conducted by using a PHI5000 Versa Probe III, Tokyo, Japan. Phase purity and the crystalline nature of the nanomaterials were examined by an X-ray diffractometer (Rigaku Smart Lab Mini flex 600, Tokyo, Japan) operated at 100 mA and 30 kV with a radiation source of Cu Kα. By using the SEM examination, the surface morphology and rough size of the produced nanoparticles were examined. The JEOL Model JSM, IT500, Tokyo, Japan was used to perform the SEM investigation. The elemental components of the produced NPs were examined using an EDX spectrum. We used TEM imaging to determine the solid particles’ precise size and shape. The produced N-ZnO Ps was drop coated on TEM grids with an aqueous dispersion before being incubated for 5–10 min. The samples were further stained with a water-in-phosphotungstic acid (PTA) solution at a concentration of 2%. The grids were then washed with ultra-pure water to remove the surplus PTA. High-resolution TEM (HRTEM) images were taken by placing a small volume of synthesized N-ZnO Ps suspension on a carbon-coated copper TEM grid and measurements were carried out on a JEOL 3010 1200 EX instrument (Tokyo, Japan) with an accelerating voltage of 80 kV. The Zeta potential of the prepared nanoparticles was analyzed by Litesizer 500, Anton Paar GmbH, Graz, Austria.

### 2.5. Antimicrobial Activity

The bacterium *Escherichia coli* (*E. coli*) and *Klebsiella pneumoniae* (*K. pneumonia*) were chosen for this investigation because of their pharmacological and therapeutic significance. After incubation for 24 h at 37 °C on Mueller–Hinton agar media, the antibacterial activity of N-ZnO Ps in various formulations was assayed by using the well diffusion technique [23]. In this investigation, three dilutions [(a) 10, (b) 20, and (c) 30 µL] of N-ZnO Ps colloidal solutions in wells were created in three wells and another well with streptomycin standard drug (30 µL of 1mg/mL). Each strain of bacteria (10^8^ CFU) was plated onto sterile Mueller–Hinton agar plates over an overnight period at 37 °C. 

### 2.6. Evaluation of Cytotoxicity

The cytotoxicity of N-ZnO Ps against LN-18 and HMC-3 cell lines was assessed by performing the MTT (3-(4,5-dimethylthiazol-2-yl)-2,5-diphenyltetrazolium bromide) assay. Cells were harvested by trypsinization during exponential development, counted, and seeded on a 96-well plate at a density of 1 × 10^4^ cells per well, and allowed to adhere for at least one night after harvesting. Over 24 h, the LN-18, and HMC-3 cells were exposed to various doses of N-ZnO Ps (6.25, 12.5, 25, 50, and 100μg/mL). Cells that had not been treated served as the control, while media that had not included any cells served as the blank and Doxorubicin as a positive control. After 24 h treatment, 100 μL of MTT (0.5 mg/mL) reagent was added to each well and incubated at 37 °C for 4 h. The medium was taken out, 100 μL of dimethyl sulfoxide was added, and then a microplate reader was used to measure the absorbance at 570 nm. The percentage viability was determined by applying the following formula:

$$\mathrm{Percentage}\;\mathrm{viability}\;=\frac{\left[\mathrm{Optical}\;\mathrm{density}\;\mathrm{of}\;\mathrm{test}\;\mathrm{sample}\;-\mathrm{Optical}\;\mathrm{density}\;\mathrm{of}\;\mathrm{blank}\right]}{\left[\mathrm{Optical}\;\mathrm{density}\;\mathrm{of}\;\mathrm{control}\;-\mathrm{Optical}\;\mathrm{density}\;\mathrm{of}\;\mathrm{blank}\right]}\times 100$$

### 2.7. Statistical Analysis 

Each value presented in the figures represents the mean ± SE of six individual determinations unless otherwise stated.

## 3. Results and Discussions

**Figure 2:**
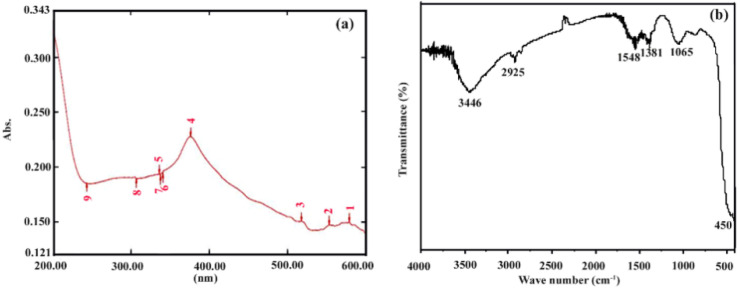
(**a**) UV-vis spectrum and (**b**)FT-IR spectrum of bio-synthesized N-ZnO Ps.

UV-Vis spectroscopy is one of the best strategies that have been used to validate the synthesis of N-ZnO Ps from green synthesized N-ZnO. When it comes to optical characteristics, N-ZnO Ps were tested using UV-visible spectroscopy. This spectrum shows the typical absorption peaks of N-ZnO Ps at a wavelength of 370 nm. The findings agree with reports in the literature as well. This is the result of the electron moving from the valence band to the conduction band. It verifies that the sample does not contain any contaminants. (Figure 2a), which is attributed to surface plasmon resonance [24,25]. FT-analysis is useful for describing functional groups present over the surface of the green synthesized N-ZnO Ps (Figure 2b). The broad peaks at around 3446 and 2925 cm^−1^ represent hydroxyl (–OH) and aldehydic (C–H) stretching vibrations, respectively [26] and it is commonly found in the ZnO. The peak at 1548 cm^−1^ may be assigned to the glycosidic linkage of C–O–C [27]. In stabilizing and capping agents, the peak at 1381 cm^−1^ represents carbonyl side-group symmetric stretching. The intense band at 1027 cm^−1^ of carbonyl (C=O) stretching vibration shows the existence of carboxylic acid groups in leaf extracts [28,29]. The region at 450 cm^−1^ in the lower energy area of the spectrum corresponds to Zn–O bond bending vibration [29].

**Figure 3:**
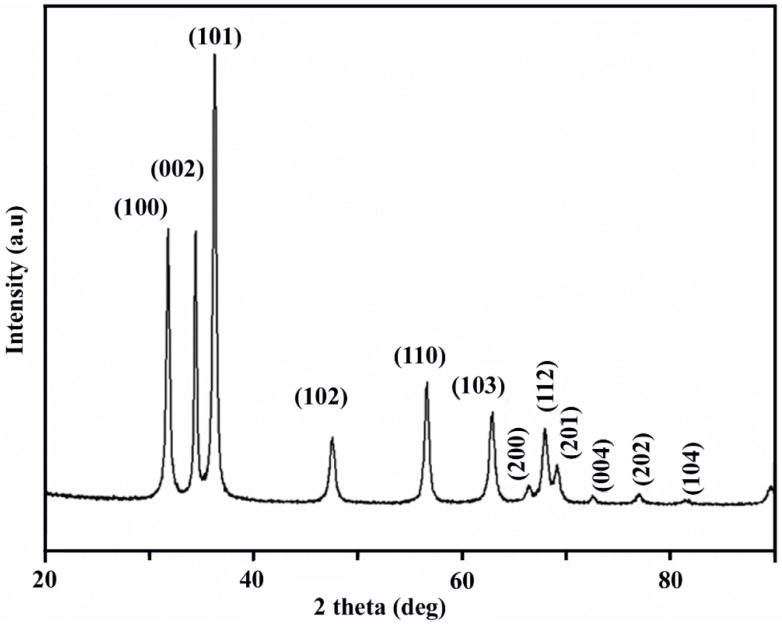
XRD pattern of N-ZnO Ps.

The XRD technique is applied to evaluate the phase purity and crystalline nature of the synthesized N-ZnO Ps obtained from perilla protein (Figure 3). The XRD patterns of N-ZnO Ps show two thetas (2θ) values at 31.74°, 34.57°, 36.42°, 47.71°, 56.79°, 63.20°, 66.57°, 68.19°, 69.40°, 72.96°, 77.13° and 81.92° (Figure 3) corresponding to (100), (002), (101), (102), (110), (103), (200), (112), (201), (004), (202), and (104) planes of the hexagonal wurtzite structure (JCPDS card no.: 36-1451) [30,31]. The size of the particle was calculated as 20 nm from the highest intensity peak using the Debye Scherrer formula [25]. The average NPs size (*D*) was evaluated with the Debye Scherrer equation:

$$D=\frac{k\lambda }{\beta cos\theta }$$

where *D* = crystallite size, *k*= shape factor generally has a value of 0.9, *λ* = wavelength of X-ray, *β* = full width at half maximum of the diffraction peak, and *θ* = Bragg diffraction angle of the diffraction peaks.

**Figure 4:**
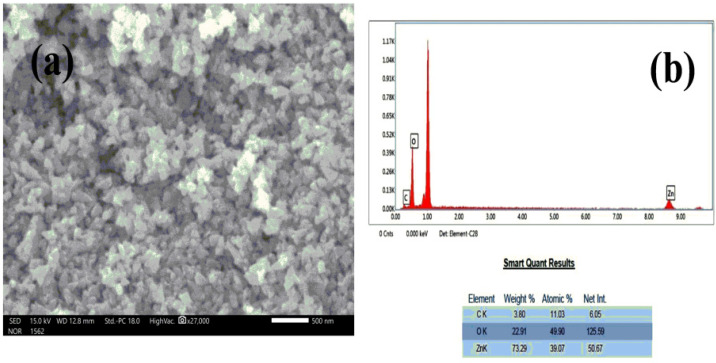
(**a**)SEM image and (**b**) EDX spectrum of N-ZnO Ps.

**Figure 5:**
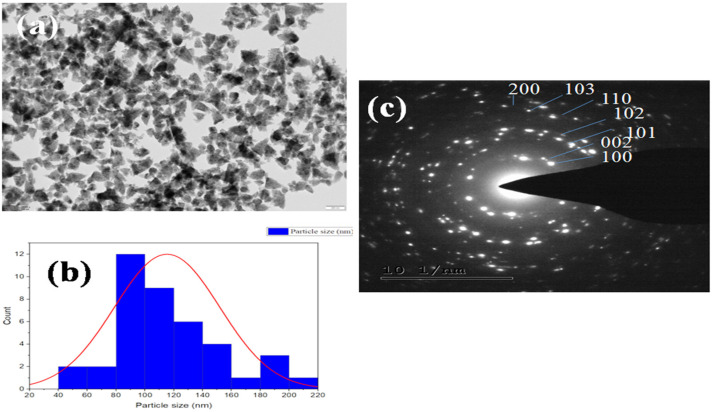
(**a**) HR-TEM images, (**b**) particle size distribution histogram of N-ZnOPs, and(**c**) SAED pattern of N-ZnO Ps.

**Figure 6:**
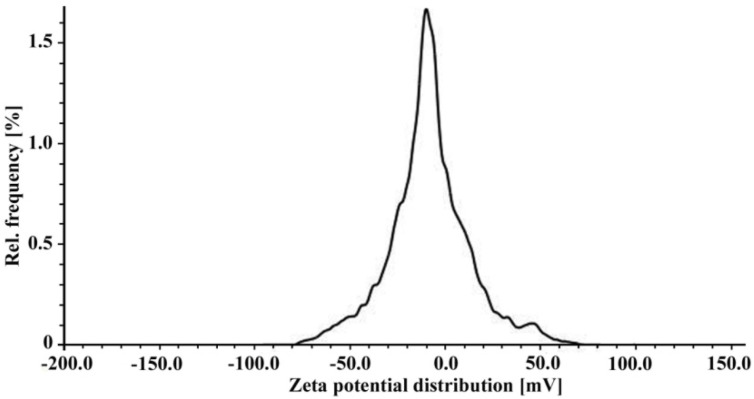
Zeta potential of the N-ZnO Ps.

The surface morphology of the green synthesized NPs was identified using the SEM examination. Figure 4 adisplays an SEM picture of the created NPs. ZnO nanoparticles have a triangular-like shape in SEM micrographs. According to the SEM micrograph, the synthesized ZnO nanoparticles were separated by a little gap between them. ZnO nanoparticles with a large surface area and tiny size are more active in several disciplines, including antibacterial activity [3]. The samples’ different elemental compositions were observed in the EDX spectra. The synthesized N-ZnO Ps showed dual peaks between 1.2 and 8.6 keV analyzed by EDX analysis (Figure 4b) and included Zn (73.29 wt.%) and O (22.91 wt.%) in addition to having peaks that indicated the presence of C. Because ZnO nanoparticles include carbon and were produced using plant material in a biogenic reduction method, there was some weight loss and a faint signal. The HR-TEM evaluation demonstrates the morphology, size, and distribution of bio-synthesized N-ZnO Ps (Figure 5). Additionally, the HR-TEM revealed a tiny aggregation that might occur as a result of the high surface energy in produced N-ZnO Ps by the extract(Figure 5a). The particle size histogram of N-ZnO Ps is presented in Figure 5b. These ZnO nanoparticles with high surface energy were created in an aqueous medium, mostly owing to the production procedure [15]. The TEM image with the SAED pattern shows that the shape is varying from triangular to polygonal with pointed ends but most of the nanoparticles are triangular-shaped with a crystalline nature (Figure 5c). The zeta potential of the prepared nanoparticles was measured as −11.3mVas, as shown in Figure 6. This can be considered strongly anionic. The zeta potential measurements thus verify and support the dispersion capacity of the green synthesized N-ZnO Ps. The negative surface charge is due to the binding affinity of extract compounds with the NPs, conferring the stability of the N-ZnO Ps and alleviating the aggregation potential of the particles [15,16].

**Figure 7:**
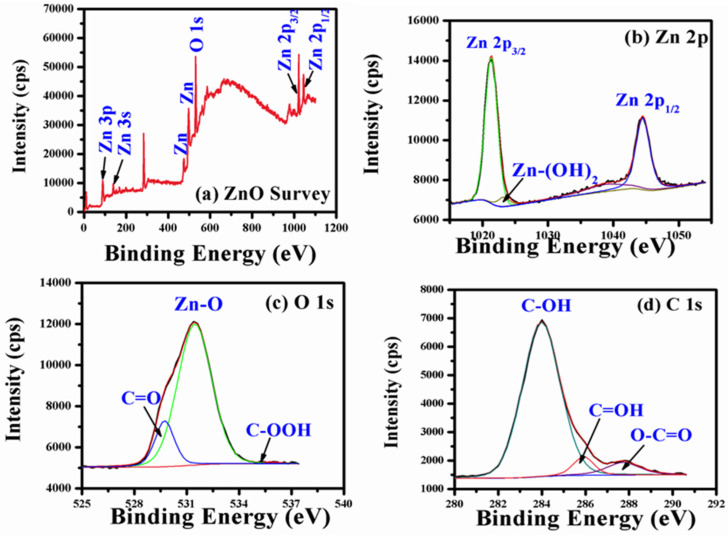
(**a**–**d**) XPS data of N-ZnO Ps.

The XPS analysis was carried out to analyze the chemical bonding nature of N-ZnO Ps prepared by the green synthesis route. Figure 7a–d illustrates the survey and high-resolution XPS spectra of N-ZnO Ps. The core-level spectral lines are associated with the Zn3p, Zn3s, O1s, and Zn2p signatures of N-ZnO Ps (Figure 7a). The additional peaks at ~475.0 and ~497.0 eV are typical Auger lines and the C 1s peak at ~284.0 eV is possibly due to residual carbon impurities on the surface of N-ZnO Ps [32,33]. The deconvoluted Zn 2p spectra reveal two strong peaks with binding energies centered at 1021.2 and 1044.4 eV, representing Zn 2p_3/2_ and Zn 2p_1/2_, respectively (Figure 7b). The obtained ~23 eV of spin-orbit splitting represents the available Zn^2+^ oxidation states in the ZnO lattice of the wurtzite structure, which is in good promise with earlier reports [33,34]. Additionally, the minor peak at ~1023.6 eV could be due to the Zn(OH)_2_ species. The deconvoluted O 1s spectrum (Figure 7c) has a primary peak at ~531.38 eV, which is attributed to the Zn–O covalent bond formation. The other minor peaks at ~529.56 and 535.1 eV could be due to surface adsorbed molecules (i.e., C=O and C–OOH) on the N-ZnO Ps, possibly situated on the surface defects [32]. Similar results are apparent in the C 1s spectrum (Figure 7d).

**Figure 8:**
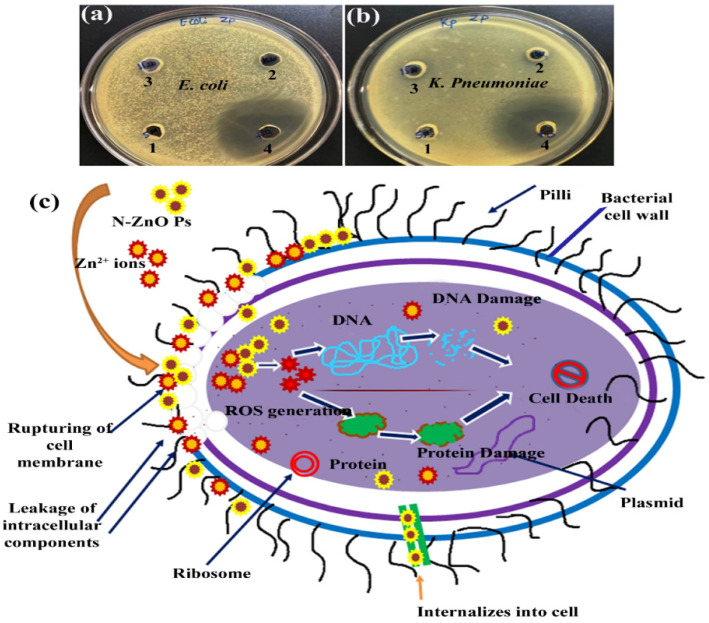
(**a**,**b**) Antibacterial activity of N-ZnO Ps against (**a**) *E. coli* and (**b**) *K.* *pneumoniae*, and (**c**) the possible antibacterial activity mechanism.

The results of antibacterial activity (ZOI) are presented in Table 1. Testing the bio-synthesized N-ZnO Ps against *E. coli* and *K. pneumoniae* reveals the effective suppressive activity of the nanoparticles (Figure 8a,b) and shows that synthesized N-ZnO Ps are useful for inhibiting bacterial growth. The bacterium *K. pneumoniae* and *E. coli* exhibited the highest ZOI, 10 ± 0.4 and 10 ± 0.5, respectively, at 30 µg/mL. This is because these small particles have a high surface-to-volume ratio compared with bulk particles, as well as superior penetrating capacity and reactivity [35]. Microorganisms become immobilized because of the interference of nanoparticles with cellular adhesion and activities at the microbial cell surface [36]. The antibacterial effect of an antibiotic is triggered by the creation of reactive oxygen species (ROS), which can damage or kill bacteria. Earlier, similar findings were observed when ZnO was activated, and electron-hole pairs increased as a result of the exposure to white light. Some of them have the potential to split H_2_O molecules into hydroxyl radicals (OH^−^) and hydrogen ions (H^+^). In some cases, the dissolved oxygen molecules are transformed into superoxide radical anions (O_2_^−^) that can subsequently combine with H^+^ to form HO^2^ radicals. This is a common occurrence. After the OH^−^ combines with electrons to form hydrogen peroxide anions H_2_O, the H_2_O_2_ molecules are formed by reacting with hydrogen to form H_2_O_2_. The H_2_O_2_ molecules produced by the bacteria penetrate the cells and destroy the bacteria [36]. The tiny size of N-ZnO Ps is demonstrated by both XRD and TEM, and these lead to a significant increase in surface area for the generation of free excitons, and consequently in the production of ROS, which is harmful to bacterial cell growth [37]. It has been demonstrated that the N-ZnOPs may attach to lipid membranes and induce disruption of the membrane structure, which can result in bacterial dysfunction, loss of membrane integrity, and eventual death [38]. The N-ZnO Ps might also enter the bacterial cells and cause toxic oxygen radical creation, thus eliminating bacterial DNA, and cellular protein, inhibiting bacterial growth and leading to bacterial death [39]. Furthermore, the antibacterial activities of the N-ZnO Ps largely occur on the surface, and thiol (–SH) group proteins contained in the bacterial cell wall structure are combined with N-ZnO Ps. This reduces cellular permeability and results in cell lysis [40,41]. The possible mechanism is presented in Figure 8c.

**Figure 9:**
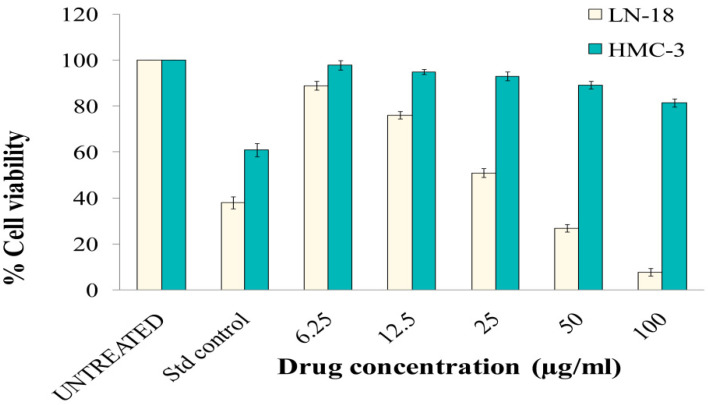
Cell viability (%) of LN-18 and HMC-3 cells treated with N-ZnO Ps.

**Figure 10:**
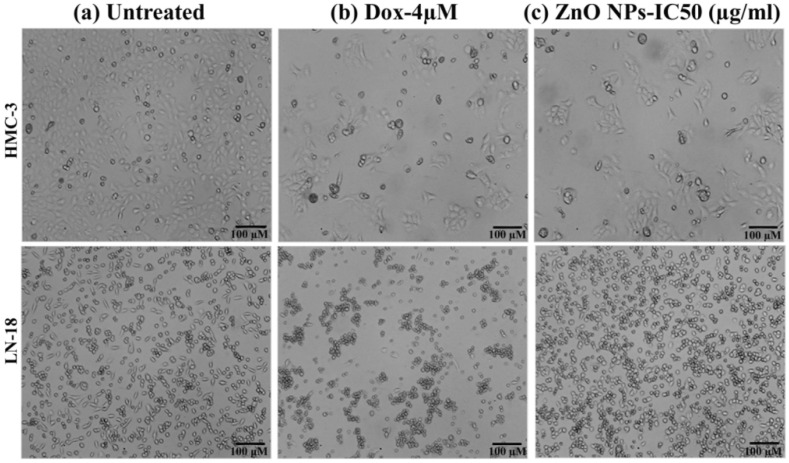
Morphology of HMC3 and LN18 cells treated with N-ZnO Ps compared: (**a**) untreated, (**b**) standard control cells, and (**c**) treated with 4 μM-doxorubicin. Scale bar 100 µm.

The cytotoxicity of N-ZnO Ps was first evaluated on cell line HMC-3 (human neuro microglia cells). The cells were treated with N-ZnO Ps at different concentrations and incubated for 24 h, in a 5% CO_2_ incubator at 37 °C. After 24 h of incubation, we observed a slight inhibition of cell proliferation of HMC-3 at the highest concentration (100 µg/mL). The viability of HMC-3 at N-ZnO Ps concentrations of 6.25, 12.5, 25, 50, and 100 µg/mL were 97%, 94%, 93%, 89%, and 81%, respectively (Figure 9). The IC_50_ value was 287.86 µg/mL, indicating lesser toxicity to these normal cells. Uncontrolled proliferation is one hallmark of cancerous cells. Further, we tested whether N-ZnO Ps had any role in inhibiting cancerous cell growth, using the LN-18 cell line (human brain glioblastoma cells). We treated LN-18 cells with different concentrations of N-ZnO Ps incubated for 24h in 5% CO_2_ at 37 °C. After 24h of incubation, N-ZnO Ps exhibited dose-dependent toxicity on LN-18 cells (Figure 10). The cell viability at N-ZnO Ps concentrations of 6.25, 12.5, 25, 50, and 100 µg/mL were 88%, 75%, 50%, 26%, and 7.6%, respectively. The IC_50_ value was 25.03 µg/mL, indicating higher cytotoxicity at a lower concentration of nanoparticles. These results indicate that N-ZnOPs particles exhibit potential antibiotic and anticancer properties and may be used in the treatment of cancer as well as infectious diseases. The main mechanism of cytotoxicity of ZnO nanoparticles is the oxidative stress generated by ROS, but this may not be the main mechanism. It is more likely that the zinc-mediated protein activity disequilibrium, a result of high intracellular zinc ions, contributes to cytotoxicity [42]. The comparison of chemical and plant extract-based N-ZnO Ps and their applications are presented in Table 2.

## 4. Conclusions

The sol-gel technique was used to create N-ZnO Ps from Perilla plant protein extract. The N-ZnO Ps were studied by UV-Vis spectrophotometer, FT-IR, HR-TEM, zeta potential, and SEM with EDX, XRD, and XPS to determine their composition. The present study demonstrated that the method of synthesizing nanoparticles using natural protein media was eco-friendly and cost-effective and that it could therefore be an economically and technologically advantageous alternative for the large-scale production of metal oxide nanoparticles in the future. The glioblastoma cell line, LN-18, and human normal brain microglia cell line, HMC-3, were both sensitive to the anticancer activity of N-ZnO Ps; however, the latter did not demonstrate any action against the HMC-3. Even with all the advantages that nanoparticles offer, in vivo applications of these particles are still rare and there is a serious lack of research on nanoparticles in vivo. A combination of ZnO NPs’ selective targeting and ability to act as a carrier agent could make them a good substitute for traditional cancer treatments. In addition, the prepared triangular-shaped nanoparticles exhibited antibacterial activity when tested with both gram-positive and negative bacteria(s).

## Figures and Tables

**Table 1:** Antibacterial activity of N-ZnO Ps.

		N-ZnOPs Suspension Zone of Inhibition (ZOI) (mm)			
S. No	Micro Organism	10 µL	20 µL	30 µL	Streptomycin 30 µL
1	*E. coli*	7 ± 1.4	9 ± 1.0	10 ± 0.4	38 ± 1.4
2	*K.* *pneumoniae*	7 ± 1.2	8 ± 1.2	10 ± 0.5	30 ± 1.7

The values are the mean of three separate trials ± SE.

**Table 2:** Comparison of chemical and green synthesis of N-ZnO Ps.

S. No	Method	Applications	Ref.
1	Chemical reduction	Antibacterial and anticancer activity	[43]
2	Co-precipitation	Antibacterial	[44]
3	Chemical reduction	Antibacterial	[45]
4	Sol-gel	Antibacterial	[46]
5	*Casuarina equisetifolia*	Antibacterial and anticancer activity	[47]
6	*Origanum vulgare*	Antibacterial and antibiofilm	[48]
7	*Perilla frutescens*	Antibacterial and neurotoxicity	Present study

## Data Availability

The data presented in this study are available on request from the corresponding author.
